# Inline mechano-vibration holography for simultaneous phase and elasticity mapping of soft samples

**DOI:** 10.1364/BOE.584264

**Published:** 2026-01-23

**Authors:** Hasan Berkay Abdioglu, Yagmur Isik, Merve Sevgi, Esmahan Caglar, Gokhan Bora Esmer, Huseyin Uvet, Ali Anil Demircali

**Affiliations:** 1 Yildiz Technical University, Department of Mechatronics Engineering, Istanbul 34349, Turkey; 2 Yildiz Technical University, Department of Bioengineering, Istanbul 34349, Turkey; 3 Yildiz Technical University, Department of Control and Automation Engineering, Istanbul 34349, Turkey; 4 Istinye University, Artificial Intelligence Research and Application Center (YZAUM), Istanbul 34467, Turkey; 5 Imperial College London, Division of Systems Medicine, Department of Metabolism, Digestion, and Reproduction, London SW7 2AZ, UK; 6 huvet@yildiz.edu.tr; 7 a.demircali@imperial.ac.uk

## Abstract

Off-axis holography enables single-shot phase retrieval but reduces spatial bandwidth, while in-line phase-shifting interferometry preserves bandwidth yet requires reference-path stepping and is sensitive to drift, limiting dynamic measurements. Moreover, viscoelastic mapping is rarely available from the same holographic measurement. We propose vibration-encoded in-line Mach-Zehnder holography for simultaneous thickness and viscoelasticity mapping of soft samples. Twelve holograms acquired over one vibration cycle are analyzed using Bessel-based harmonic inversion and robust regression to recover the static phase, modulation depth, and phase lag, yielding thickness and Kelvin-Voigt storage and loss modulus maps (*E*′, *E*″). Simulations recover *E*′ and *E*′′ to within ∼2% across a wide *E*′′/*E*′ range and achieve sub-micron thickness error over 20-45 *μ*m beads. Experiments on calibrated polyacrylamide beads show sub-micron thickness repeatability (median ∼0.57 *μ*m over 40 repeats) and stiffness estimates typically within 10% of ground truth, and we further demonstrate the approach on adherent MCF-7 cells.

## 1. Introduction

Quantitative analysis of cells and tissues is fundamental for understanding their morphology, dynamics, and mechanical properties, all of which are closely linked to physiological and pathological processes [1–5]. Mechanical analysis relies on inducing a controlled deformation or vibration in the sample and measuring its mechanical response as displacement, from which stiffness or elasticity can be inferred [4,6]. Morphological analysis, in contrast, quantifies the refractive-index and thickness variations encoded in the optical phase, which together describe the three-dimensional geometry of cells. These parameters jointly reflect cellular elasticity, deformation, and migration, serving as key indicators of biological function and disease progression [1,5].

Among optical modalities for morphological assessment, digital holographic microscopy (DHM) has proven particularly powerful, enabling full-field, label-free visualization of transparent specimens with sub-wavelength sensitivity [7,8]. DHM reconstructs the optical phase distribution of a sample, which encodes both its refractive-index and thickness variations. However, the phase information cannot be measured directly from a single hologram. Several strategies have been developed to retrieve the phase information, each with its own advantages and limitations [9,10]. For instance, in inline holography, the reference and object waves share the same optical path, producing a compact and stable setup. However, since the real image, virtual image (twin image) and DC terms overlap in the recorded intensity, phase retrieval typically requires additional techniques such as Phase-Shifting Interferometry (PSI) [11–13]. In PSI, a controlled phase delay is introduced by elongating the reference path to numerically separate these overlapping terms [12,14,15]. The object must remain perfectly stationary during phase shifting, which makes it unsuitable for moving or dynamic samples. Moreover, PSI systems are highly sensitive to environmental instabilities such as air fluctuations or mechanical vibrations; even slight deviations from the intended phase step can introduce significant reconstruction errors [16]. At the same time, inline phase retrieval can also be performed from a single measurement using iterative phase-retrieval or regularized inverse-problem formulations; however, these approaches typically rely on strong priors or constraints to suppress twin-image/DC ambiguities and to stabilize the solution, which can bias reconstructions when experimental conditions deviate from assumptions [17,18].

For mechanical characterization, gold-standard techniques such as Atomic Force Microscopy (AFM) and optical tweezers have been widely employed to measure the stiffness of biological samples at the single-cell level. AFM achieves nanometer-scale precision by indenting the cell membrane with a calibrated probe, allowing localized elasticity mapping and detection of subcellular mechanical variations [19–21]. However, despite its accuracy, AFM is inherently slow, requires complex calibration, and can introduce artifacts due to probe–sample contact, tip geometry, or substrate coupling [22]. These limitations restrict its applicability for high-throughput or dynamic measurements. Optical tweezers, on the other hand, enable noncontact manipulation of cells using photon momentum, offering piconewton force sensitivity [23–25]. Nevertheless, they demand highly stable optical alignment, expensive high–numerical-aperture objectives, and are best suited for suspended cells rather than adherent populations commonly used in mechanobiology [26].

At the intersection of digital holography and mechanical/vibration analysis, dynamic holographic methods have long been used to visualize deformation and extract vibration signatures from reconstructed wavefields. A representative example is phase-shifting holographic vibration analysis, where multiple phase-stepped holograms are combined to recover the complex field and produce Bessel-fringe patterns that encode vibration amplitude as contour maps [27,28]. While powerful for vibration metrology, these approaches are primarily tailored to amplitude mapping (often under time-averaged recording) and typically do not extend to quantitative mechanical property estimation (e.g., elastic modulus) of the interrogated objects.

To address these needs in soft-matter and mechanobiology settings, we previously demonstrated a noninvasive off-axis holographic sensor system capable of estimating the stiffness of soft microscopic samples by capturing vibration-induced phase modulations generated by a piezoelectric transducer [29]. By comparing phase maps acquired at different vibration states and fitting a Hertzian mechanical model, we quantified the elastic modulus of the samples. However, the off-axis configuration required precise alignment, high computational demand, angular separation, and spatial filtering, which reduced usable spatial bandwidth and limited optical throughput.

To overcome these limitations and extend the mechanical analysis beyond purely elastic behavior, we developed a Mach-Zehnder interferometric inline holographic system integrated with a piezoelectric transducer for mechano-vibration-based phase and viscoelastic characterization. In this configuration, a soft sample (e.g., a polyacrylamide microbead or a biological cell) is driven sinusoidally, and 12 sequential holograms are recorded within a single vibration cycle. The periodic motion induces intrinsic, object-localized phase modulation, from which we recover the quantitative phase map and simultaneously estimate viscoelastic parameters by fitting a Kelvin-Voigt model. Compared to off-axis holography, the proposed inline approach preserves the full spatial bandwidth by avoiding angular separation, thereby improving both resolution and field of view. Relative to classical PSI, it eliminates the need for externally controlled phase plates or synchronized modulators. Importantly, while single-shot iterative or regularized inline phase-retrieval approaches can be applied to dynamic scenes, they typically rely on strong priors or constraints to suppress the twin-image and DC terms and to stabilize the reconstruction [17,18]. In contrast, our method exploits actuation-induced phase changes that are intrinsically localized to the object region, enabling pixel-wise phase and displacement recovery without imposing restrictive reconstruction priors. Overall, this configuration provides a robust framework for quantitative evaluation of both morphological information and viscoelastic mechanical properties of soft microscopic materials.

## 2. Methods and materials

### 2.1. Simulation algorithm

Synthetic inline hologram sequences were generated in MATLAB under controlled optical conditions, including a defined optical passband and a prescribed reference and object wavefront mismatch. A circular phase object representing a soft microsphere of diameter D=45μm and refractive-index contrast $\Delta n=n_{\text{bead}}-n_{\text{medium}}=1.39-1.34=0.05$ was placed in a water-like medium. Illumination wavelength was λ=671nm , the sensor pixel pitch $p_{s}=4.7\;\mu \text{m}$ , and magnification M=20× , yielding an object-plane sampling of $p_{o}=p_{s}/M=0.235\;\mu \text{m}$ . The field of view was N×N=1024×1024 pixels. To reflect realistic bandwidth limits, the object field was filtered using a circular pupil corresponding to a numerical aperture NA=0.4 :

(1) $$f_{\text{cut}}=\frac{\mathrm{NA}}{M\;\lambda },$$

applied in the spatial-frequency domain using an angular-spectrum (Fourier) propagation step. A mild curvature mismatch between the object and reference waves was introduced to emulate imperfect optical alignment. The corresponding quadratic phase terms were defined as

(2) $$Q_{\text{obj}}(x,y)=\exp\;\left[j\;\frac{k}{2R_{\text{obj}}}(x^{2}+y^{2})\right],\;Q_{\text{ref}}(x,y)=\exp\;\left[j\;\frac{k}{2R_{\text{ref}}}(x^{2}+y^{2})\right],$$

with $R_{\text{obj}}=0.080\;\text{m}$ , $R_{\text{ref}}=0.079\;\text{m}$ , and k=2π/λ . No other aberrations or random phase fluctuations were introduced.

The static optical phase was given by

(3) $$\phi_{0}(x,y)=k\;\Delta n\;h(x,y),$$

where the spherical thickness profile inside the bead was

(4) $$h(x,y)=2\sqrt{R_{\text{bead}}^{2}-r^{2}},\;r=\sqrt{x^{2}+y^{2}}.$$

Object vibration at f=10Hz produced a time-varying phase modulation

(5) $$\phi (x,y,t)=\phi_{0}(x,y)+\beta (x,y)\sin(\omega t-\delta (x,y)),\;\omega =2\pi f,$$

where β(x,y) denotes the local phase modulation depth induced by the vibration amplitude of the object and δ(x,y) is the local phase lag between the applied sinusoidal excitation and the measured response (Kelvin–Voigt viscoelasticity). Physically, *β* represents the peak phase excursion at each pixel caused by the periodic axial displacement of the sample and is proportional to the product of the optical wavenumber *k* and the local vibration amplitude Δz(x,y) , i.e.,

(6) β(x,y)=kΔz(x,y).

Each hologram was computed by combining the object and reference fields as

(7) $$\begin{array}{ll} O(x,y;\theta_{m}) & =A_{\text{obj}}(x,y)\;Q_{\text{obj}}(x,y)\;\exp\;[j\;\phi (x,y,\theta_{m})],\\ R(x,y) & =A_{\text{ref}}\;Q_{\text{ref}}(x,y), \end{array}$$

where $A_{\text{obj}}(x,y)$ denotes the (real-valued) object-field amplitude envelope at the camera plane, $A_{\text{ref}}$ is the reference-wave amplitude (assumed spatially uniform), and $\theta_{m}$ denotes the sampling phase of the *m*th hologram within one vibration period (i.e., the instantaneous vibration state at which the hologram is recorded). For uniform sampling of a sinusoidal excitation over one cycle, we set

(8) $$\theta_{m}=\frac{2\pi (m-1)}{N},\;m=1,\ldots ,N,$$

where *N* is the number of holograms acquired per vibration cycle (here N=12 ).

In the simulations, the object and reference field amplitudes were normalized to unit magnitude, with $A_{\mathrm{obj}}(x,y)=1$ and $A_{\mathrm{ref}}=1$ . Accordingly, the time-varying phase in Eq. (5) is sampled as $\phi (x,y,\theta_{m})=\phi_{0}(x,y)+\beta (x,y)\sin(\theta_{m}-\delta (x,y))$ . The resulting intensity hologram sequence was synthesized as

(9) $$I_{m}(x,y)={\left|O(x,y;\theta_{m})+R(x,y)\right|}^{2}.$$

where *O* accounts for the object curvature and the NA-imposed band-limit, and *R* accounts for the reference-wave curvature. No external phase or angular jitter was applied; the quantitative impact of phase/angle jitter on the recovered parameters is instead evaluated separately in Supplement 1 Table S1 and S2. All simulated holograms were saved as globally scaled 16-bit TIFF images. For the viscoelastic ground truth, spatial maps of Kelvin-Voigt parameters were generated within the bead region, namely the elastic storage modulus $E_{\mathrm{GT}}(x,y)$ and viscous (loss) modulus $\eta_{\mathrm{GT}}(x,y)$ . Under sinusoidal excitation, the complex modulus magnitude is

(10) $$|E_{\text{GT}}^{*}(x,y)|=\sqrt{{\left(E_{\text{GT}}(x,y)\right)}^{2}+{\left(\omega \eta_{\text{GT}}(x,y)\right)}^{2}},$$

and the phase lag is

(11) $$\delta (x,y)=\delta_{\text{GT}}(x,y)=\mathrm{atan}2\;\left(\omega \;\eta_{\text{GT}}(x,y),\;E_{\text{GT}}(x,y)\right).$$

For an applied stress amplitude $\sigma_{\text{peak}}=100\;\text{Pa}$ , the local strain amplitude is

(12) $$\varepsilon (x,y)=\frac{\sigma_{\text{peak}}}{\left|E_{\text{GT}}^{*}(x,y)\right|},$$

which sets the phase-modulation depth through

(13) $$\beta (x,y)=\varepsilon (x,y)\;\phi_{0}(x,y).$$

The simulations were performed under deterministic optical constraints, including numerical aperture band-limiting, a prescribed wavefront curvature mismatch, and controlled viscoelastic heterogeneity, with measurement noise excluded.

### 2.2. Reconstruction algorithm

**Fig. 1.**
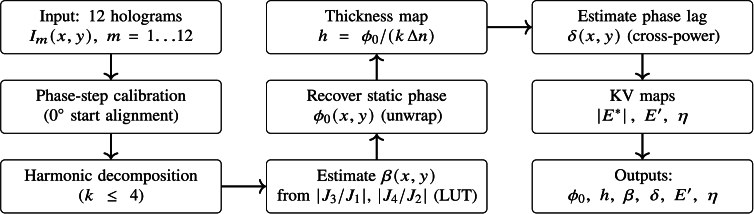
Overview of the reconstruction pipeline. A 0^∘^ start alignment is first applied to each 12-frame cycle, followed by harmonic analysis to recover *β*, $\phi_{0}$ , *δ*, and Kelvin–Voigt viscoelastic maps.

A reconstruction pipeline was implemented to extract quantitative phase, thickness, and Kelvin–Voigt viscoelastic parameter maps from 12-step vibration sequences. During vibration, twelve sequential holograms corresponding to equally spaced vibration phases were recorded as intensity images. The main stages of the reconstruction procedure were summarized in Fig. 1.

#### (a) Phase-step calibration (0^∘^ start alignment)

In practice, the first recorded frame of each 12-image cycle may not correspond to the same vibration phase due to ordering offsets. Therefore, before harmonic analysis, each cycle is circularly shifted by an integer index s∈{0,…,11} to enforce a consistent 0^∘^ start. The shift is estimated using a reference region outside the object, which is driven together with the sample but does not contain the object-specific, localized phase modulation. To make the criterion insensitive to the unknown static phase $\phi_{0}(x,y)$ , we form cross-power phasors from normalized harmonics (defined in Sec. (b)):

(14) $$D_{31}(x,y)=h_{3}(x,y)\;h_{1}^{*}(x,y),\;D_{42}(x,y)=h_{4}(x,y)\;h_{2}^{*}(x,y),$$

and compute a weighted average over the reference mask. The cycle shift *s* is selected by a discrete search over s=0,…,11 to align the reference phasor to a consistent 0^∘^ direction, and the 12 frames are circularly shifted accordingly. This step ensures that all cycles share the same phase-step ordering prior to accumulation.

#### (b) Harmonic decomposition and phase–modulation extraction

During vibration, each pixel experiences a time-varying optical phase (Eq. (5)). The temporal sequence across vibration phases $\theta_{m}=2\pi (m-1)/M$ contains a periodic variation that can be expressed as a discrete Fourier series. The complex harmonic coefficient at order *k* is estimated by

(15) $$C_{k}(x,y)=\frac{1}{M}\sum_{m=1}^{M}I_{m}(x,y)\;e^{-jk\theta_{m}},\;k=0,1,2,3,4,$$

where M=12 and $C_{0}(x,y)$ corresponds to the per-pixel DC term. In implementation, we use the normalized harmonics $h_{k}(x,y)=C_{k}(x,y)/\max(C_{0}(x,y),\epsilon )$ to reduce sensitivity to slow intensity scaling. Harmonic orders up to k=4 were analyzed because sinusoidal phase modulation expands into multiple harmonics whose amplitudes depend on $J_{k}(\beta )$ , while retaining a compact computational footprint.

Under sinusoidal phase modulation with a lag, the phase term expands as

(16) $$e^{\;j\beta \sin(\theta -\delta )}=\sum_{k=-\infty }^{\infty }J_{k}(\beta )\;e^{\;jk(\theta -\delta )},$$

where $J_{k}(\beta )$ is the *k*-th order Bessel function of the first kind. This shows that temporal harmonics have magnitudes governed by $\left|J_{k}(\beta )\right|$ and phase factors governed by *δ*.

Direct use of the harmonic magnitudes is unreliable because their absolute values depend on illumination intensity and reflectivity. To remove this dependency, we form normalized ratios between higher-order and lower-order harmonics,

(17) $$r_{31}(x,y)=\frac{A_{3}}{A_{1}}\approx \frac{\left|J_{3}(\beta )\right|}{\left|J_{1}(\beta )\right|},\;r_{42}(x,y)=\frac{A_{4}}{A_{2}}\approx \frac{\left|J_{4}(\beta )\right|}{\left|J_{2}(\beta )\right|},$$

where $A_{k}(x,y)=|h_{k}(x,y)|$ . Over the first lobe, $r_{31}$ and $r_{42}$ vary monotonically with *β*, enabling direct numerical inversion. In this work, inversion is implemented using a lookup table (LUT) generated numerically from Eq. (17) by evaluating the Bessel functions $J_{k}(\cdot )$ [30]: we sample *β* on a dense grid $\beta_{i}\in [0,\beta_{\max}]$ , compute $R_{31}(\beta_{i})=|J_{3}(\beta_{i})|/|J_{1}(\beta_{i})|$ and $R_{42}(\beta_{i})=|J_{4}(\beta_{i})|/|J_{2}(\beta_{i})|$ , and recover $\hat{\beta }(x,y)$ by 1D interpolation of the monotonic mapping over the first lobe.

Finally, the odd- and even-ratio inversions (from $r_{31}$ and $r_{42}$ ) are combined to obtain a single $\hat{\beta }(x,y)$ estimate.

#### (c) Static phase retrieval

The static optical phase $\phi_{0}(x,y)$ is recovered from the harmonic terms after estimating β(x,y) and applying the corresponding Bessel factors. A wrapped phase estimate is formed and then unwrapped using a Transport-of-Intensity-Equation (TIE)-based method. The unwrapped phase is referenced using a background (glass) region by subtracting its median value; optionally, a low-order plane is removed from the glass region before referencing to compensate residual curvature mismatch.

#### (d) Phase lag estimation δ(x,y)

The local phase lag is estimated using cross-power products that cancel the unknown static phase while preserving the *δ*-dependent factors. Using the normalized harmonics $h_{k}$ , we compute $D_{31}=h_{3}h_{1}^{*}$ and $D_{42}=h_{4}h_{2}^{*}$ and combine them with magnitude-based weights to obtain a single complex phasor D(x,y) . The phase lag map is then obtained as

(18) $$\hat{\delta }(x,y)=\left|\;-\frac{1}{2}∠D(x,y)\right|,$$

and values are constrained to the physically relevant interval [0,π/2] .

#### (e) Thickness and Kelvin–Voigt viscoelastic mapping

The quantitative thickness distribution is computed from the unwrapped phase as

(19) $$h(x,y)=\frac{\lambda \;\phi_{0}(x,y)}{2\pi \;\Delta n},$$

where *λ* is the illumination wavelength and $\Delta n=n_{\mathrm{object}}-n_{\mathrm{medium}}$ is the refractive-index contrast. For viscoelastic analysis, the strain amplitude follows from the matched relation $\beta =\varepsilon \;\phi_{0}$ ,

(20) $$\varepsilon (x,y)=\frac{\beta (x,y)}{\phi_{0}(x,y)}.$$

Assuming a sinusoidal stress field of amplitude *σ* generated by the piezoelectric transducer, the complex modulus magnitude is

(21) $$\left|E^{*}(x,y)\right|=\frac{\sigma }{\varepsilon (x,y)}=\frac{\sigma \;\phi_{0}(x,y)}{\beta (x,y)}.$$

Here, the stress amplitude *σ* in the sample plane is obtained from finite-element simulations of the transducer-chip configuration in COMSOL Multiphysics under the same excitation conditions (for details, see [29]). Using the estimated phase delay δ(x,y) , the Kelvin–Voigt storage and loss moduli are

(22) $$E^{\prime }(x,y)=\left|E^{*}(x,y)\right|\;\cos\delta (x,y),\;E^{\prime \prime }(x,y)=\left|E^{*}(x,y)\right|\;\sin\delta (x,y),$$

and the viscosity is obtained as

(23) $$\eta (x,y)=\frac{E^{\prime \prime }(x,y)}{\omega },$$

where ω=2πf is the angular frequency of actuation.

### 2.3. Production of transducer-integrated PDMS chip

A transducer-integrated poly(dimethylsiloxane) (PDMS) chip was fabricated. The chip geometry was designed in SolidWorks, and fabrication was performed using soft lithography with an aluminum mold measuring 24 mm × 60 mm × 10 mm (length × width × height). Poly(dimethylsiloxane) base and curing agent (Sylgard 184, Dow Corning) were mixed at a 10:1 mass ratio, poured into the mold, degassed in a desiccator for 45 min, and cured at 65^∘^C for 3 h. After curing, the PDMS layer was released from the mold and trimmed to the required dimensions. The PDMS substrate was bonded to a piezoelectric transducer (PL122.10, Physik Instrumente, Germany) using oxygen plasma treatment at 600 mTorr for 50 s. The integrated chip was mounted on a three-axis positioning stage within the interferometric microscope and used as the sample holder for all measurements.

### 2.4. Optical measurement setup

**Fig. 2.**
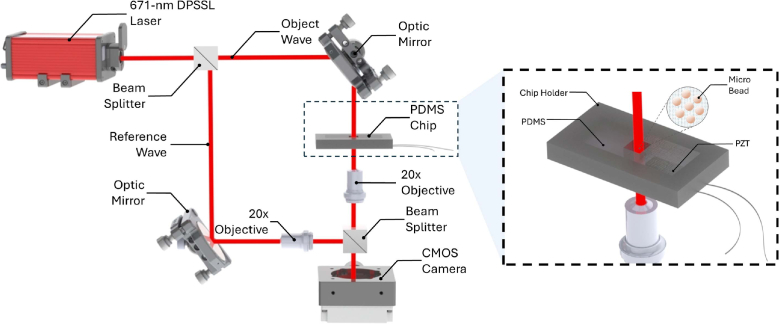
Schematic of the in-line Mach–Zehnder interferometric setup and the transducer-integrated microchip. A 671-nm DPSSL laser beam is divided by a 50/50 beam splitter into reference and object waves. Both arms are guided through 20× microscope objectives and mirrors before recombining at the detection beam splitter, where the interference pattern is recorded on a CMOS camera. The inset illustrates the PDMS-based chip integrated with a piezoelectric (PZT) transducer, which vibrates the sample (microbeads or cells) during image acquisition. This configuration enables the acquisition of multiple phase-shifted holograms within a single vibration cycle without mechanical path modulation.

The experimental setup was based on an in-line Mach–Zehnder interferometric configuration designed to capture phase-resolved holograms during controlled mechanical excitation (see Fig. 2. A single-longitudinal-mode (SLM) diode-pumped solid-state laser operating at a wavelength of λ=671nm and an output power of 200 mW was employed as the illumination source. The laser provided a coherence length exceeding 50 m, ensuring stable interference and minimal temporal phase noise. The object and reference beams were coupled coaxially using a non-polarizing beam splitter and recombined at the image plane of the microscope objective to form an in-line hologram on the camera sensor.

Image acquisition was performed using a Basler boost boA1936–400cm camera (4.5 μm pixel size). During each measurement, the sample was vibrated sinusoidally at a frequency of 100 Hz by the integrated piezoelectric bender actuator. The transducer was driven by a 20 V_pp_ sinusoidal signal generated by a Siglent SDG-series function generator.

The camera operated at 1200 frame per second (fps), recording twelve sequential holograms over a 0.01 s interval-corresponding precisely to one full vibration cycle. This acquisition rate yielded an effective temporal phase increment of $30^{\circ }$ between consecutive frames, consistent with the simulation conditions. The incident optical power on the sample was adjusted to maintain high interference contrast while avoiding photothermal effects. All holograms were processed using the reconstruction pipeline described in Sections (b)–(d) to obtain quantitative phase, thickness, and viscoelastic maps.

## 3. Results

### 3.1. Simulation results

The proposed reconstruction pipeline was quantitatively validated using numerically generated in-line holograms of harmonically vibrating microspheres simulated in MATLAB. In all simulations, N=12 phase-shifted holograms were sampled uniformly over one vibration period at $30^{\circ }$ intervals (λ=671nm,M=20,NA=0.4) , providing a practical balance between temporal sampling density and reconstruction stability. The forward model included a mild curvature mismatch between the object and reference waves and an NA-limited pupil filter to emulate the imaging system band limit. The sample mechanics followed a Kelvin–Voigt (KV) linear viscoelastic model parameterized by storage and loss moduli, $E^{\prime }(x,y)$ and $E^{\prime \prime }(x,y)$ (with $E^{\prime \prime }=\omega \eta$ ). No additional measurement noise was added; the only stochastic component was a spatially smooth random texture imposed on the ground-truth mechanical maps to mimic realistic heterogeneity.

**Fig. 3.**
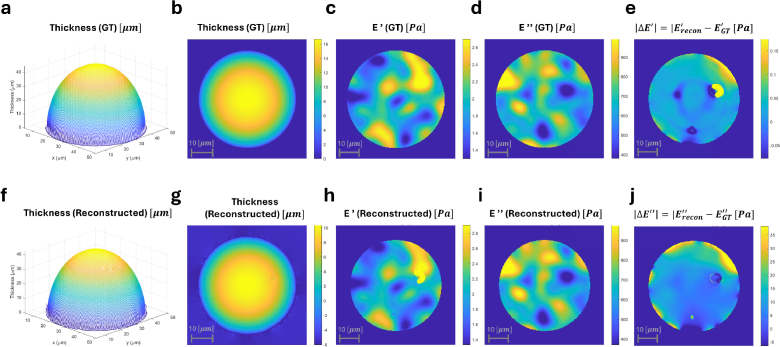
Simulation results (ground truth vs. reconstruction). (a) Thickness (ground truth) 3D mesh; (b) Thickness (ground truth) 2D map; (c) Storage modulus $E^{\prime }$ (ground truth) map; (d) Loss modulus $E^{\prime \prime }$ (ground truth) map; (e) Absolute difference in storage modulus, $\left|\Delta E^{\prime }|=|E_{\mathrm{recon}}^{\prime }-E_{\mathrm{GT}}^{\prime }\right|$ . (f) Thickness (reconstructed) 3D mesh; (g) Thickness (reconstructed) 2D map; (h) Storage modulus $E^{\prime }$ (reconstructed) map; (i) Loss modulus $E^{\prime \prime }$ (reconstructed) map; (j) Absolute difference in loss modulus, $\left|\Delta E^{\prime \prime }|=|E_{\mathrm{recon}}^{\prime \prime }-E_{\mathrm{GT}}^{\prime \prime }\right|$ . Scale bars: 10 μm.

After phase retrieval, the reconstructed thickness was referenced to an edge-zeroed baseline determined by a rim-percentile criterion, and β(x,y) was stabilized using a mild regularization (odd-harmonic weighting near the rim followed by median filtering and clamping) to suppress boundary artifacts; the quantitative impact of these steps is summarized in Supplement 1 Table S3. Representative ground-truth and reconstructed maps are shown in Fig. 3. The top row presents (a,b) the ground-truth thickness in 3D and 2D, (c,d) the ground-truth KV maps ( $E^{\prime }$ and $E^{\prime \prime }$ ), and (e) the absolute $E^{\prime }$ error map $\left|\Delta E^{\prime }|=|E_{\mathrm{recon}}^{\prime }-E_{\mathrm{GT}}^{\prime }\right|$ . The bottom row shows the corresponding reconstructed quantities (f–i) and the absolute $E^{\prime \prime }$ error map (j), $\left|\Delta E^{\prime \prime }|=|E_{\mathrm{recon}}^{\prime \prime }-E_{\mathrm{GT}}^{\prime \prime }\right|$ .

Across the simulated bead diameter range (D=20–45μm), the reconstructed thickness profiles agreed well with the geometric ground truth, with thickness RMSE values remaining submicron to low-micron depending on bead size (Table 2). The reconstructed thickness surfaces preserve the expected spherical-cap geometry and boundary definition (Fig. 3(f),g), confirming that the phase referencing strategy maintains quantitative thickness contrast.

The KV mechanical reconstructions showed similarly high fidelity. The recovered storage modulus maps $E_{\mathrm{recon}}^{\prime }(x,y)$ and loss modulus maps $E_{\mathrm{recon}}^{\prime \prime }(x,y)$ reproduce the imposed spatial patterns of $E_{\mathrm{GT}}^{\prime }(x,y)$ and $E_{\mathrm{GT}}^{\prime \prime }(x,y)$ (Fig. 3(c),d versus h,i). The absolute error maps (Fig. 3(e),j) indicate that residual discrepancies are mainly localized near the rim, while the central region remains uniform and quantitatively consistent with the ground truth.

To quantify performance in the viscoelastic regime, we fixed the ground-truth storage modulus at $E_{\mathrm{GT},\mathrm{mean}}^{\prime }\approx 2002.5\;\text{Pa}$ and swept the loss-to-storage ratio $ratio=E^{\prime \prime }/E^{\prime }$ (12 phases, 10 Hz). As summarized in Table 1, the reconstructed $E_{\mathrm{rec},\mathrm{mean}}^{\prime }$ remained close to the target value across the full ratio range, and $E_{\mathrm{rec}}^{\prime \prime }$ tracked the prescribed $E_{\mathrm{GT}}^{\prime \prime }$ with small deviation. The reconstructed complex modulus magnitude ${\left|E^{*}\right|}_{\mathrm{rec}}$ followed the expected monotonic increase with ratio, demonstrating that the pipeline preserves both the absolute scale and elastic–viscous trends imposed by the KV model.

**Table 1.** Fixed storage modulus $E_{\mathrm{GT}}^{\prime }\approx 2002.5$ Pa, sweep of $E^{\prime \prime }/E^{\prime }$ , loss-to-storage ratio, (12 phases, 100 Hz).

$E^{\prime \prime }/E^{\prime }$	$E_{\mathrm{GT}}^{\prime \prime }(\text{Pa})$	${\left|E^{*}\right|}_{\mathrm{GT}}(\text{Pa})$	$E_{\mathrm{rec},\mathrm{mean}}^{\prime }(\text{Pa})$	$E_{\mathrm{rec}}^{\prime \prime }(\text{Pa})$	${\left|E^{*}\right|}_{\mathrm{rec}}(\text{Pa})$
0.05000	100.1	2005.0	2033.8	101.7	2036.3
0.12959	259.5	2019.2	2030.7	263.2	2047.7
0.20918	418.9	2045.8	2039.1	426.5	2083.2
0.28878	578.3	2084.4	2039.1	588.2	2122.3
0.36837	737.6	2134.0	2042.2	752.2	2176.3
0.44796	897.0	2194.1	2042.9	914.5	2238.4
0.52755	1056.4	2263.9	2041.7	1076.8	2308.3
0.60714	1215.7	2342.8	2032.4	1233.0	2377.3
0.68673	1375.1	2429.3	2034.2	1396.5	2467.7
0.76633	1534.5	2522.3	2037.9	1559.6	2566.2
0.84592	1694.0	2620.5	2041.4	1723.0	2671.4
0.92551	1853.4	2723.7	2044.3	1884.6	2780.5

We also evaluated geometric scaling by fixing the viscosity at η=2.5 (12 phases, 100 Hz) and sweeping bead diameter D=20–45μm (Table 2). The reconstructed thickness mean values remained tightly centered on the ground-truth diameters and the thickness RMSE stayed within a low-micron envelope across all sizes. The reconstructed elasticity statistics were stable with respect to diameter, indicating consistent recovery across changing curvature and masked area.

**Table 2.** Fixed viscosity η=2.5 (12 phases, 100 Hz) measured for beads of varying diameters (D=20–45μm).

D(µm)	$E_{\mathrm{mean}}(\text{Pa})$	$E_{\mathrm{RMSE}}(\text{Pa})$	$T_{\mathrm{GT}}(\text{µm})$	$T_{\mathrm{mean}}(\text{µm})$	$T_{\mathrm{RMSE}}(\text{µm})$	$N_{\mathrm{pix}}$
20	1513.70	28.28	20.00	20.02	0.23	2765
25	1625.70	138.64	25.00	24.98	0.60	4773
30	1605.50	112.32	30.00	29.93	0.37	7369
35	1641.10	160.61	35.00	34.99	0.92	10493
40	1646.00	155.39	40.00	40.01	0.55	14133
45	1630.60	150.39	45.00	44.95	1.06	18393

Overall, the simulations confirm that the proposed in-line framework can recover thickness and KV viscoelastic parameters ( $E^{\prime }$ and $E^{\prime \prime }$ ) from one vibration cycle of phase-stepped holograms under ideal, noise-free conditions, providing a controlled baseline for the subsequent experimental validation on calibrated polyacrylamide beads.

### 3.2. Validation on polyacrylamide beads

To verify the quantitative accuracy and repeatability of the proposed in-line holographic reconstruction framework, thickness and viscoelastic parameters were first measured on calibrated polyacrylamide beads. Ten distinct beads were imaged, and for each bead 40 repeated measurements were acquired under identical vibration and illumination conditions. A geometric ground-truth (GT) thickness for each bead was computed from its lateral diameter (spherical approximation) and used as an independent reference for the reconstructed thickness.

**Fig. 4.**
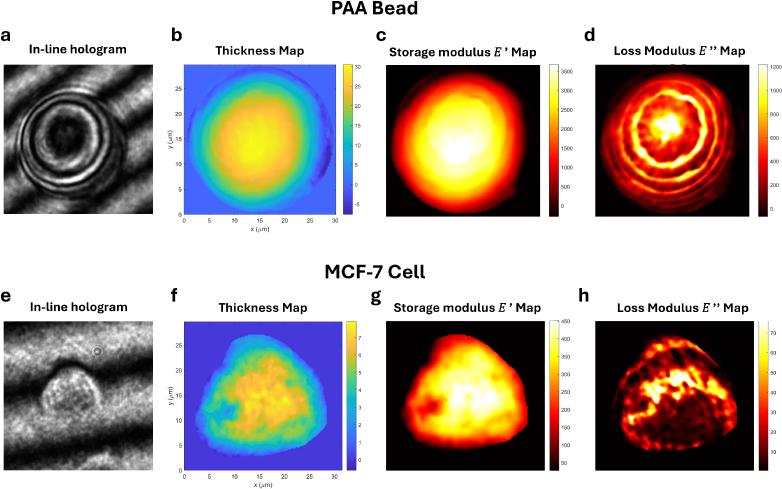
Experimental results. Top row (PAA bead): (a) in-line hologram (raw bead holograms are provided in Dataset 1 [31]); (b) thickness (2D map); (c) storage modulus $E^{\prime }$ map; (d) loss modulus $E^{\prime \prime }$ map. Bottom row (MCF-7 cell): (e) in-line hologram; (f) thickness (2D map); (g) storage modulus $E^{\prime }$ map; (h) loss modulus $E^{\prime \prime }$ map. Axes are in μm; scale bars: 10 μm.

Representative experimental reconstructions are shown in Fig. 4. The raw bead hologram sequence used for Fig. 4(a) is provided as Dataset 1 [31]. For the bead example (top row), the raw in-line hologram is shown in Fig. 4(a). The reconstructed thickness map in Fig. 4(b) exhibits the expected smooth spherical-cap morphology. The corresponding Kelvin–Voigt parameter maps, $E^{\prime }$ and $E^{\prime \prime }$ , are shown in Fig. 4(c) and Fig. 4(d), respectively.

Quantitative results for all beads are summarized in Table 3. The reconstructed thickness values agree well with the corresponding geometric GT values across the dataset, demonstrating that the phase-to-thickness conversion is quantitatively consistent under experimental conditions. The reconstructed storage modulus values span $E_{\mathrm{mean}}^{\prime }=1129–2247\;\text{Pa}$, while the loss modulus values span $E^{\prime \prime }=136–515\;\text{Pa}$. Averaged across the 10 beads, the mean reconstructed storage modulus was $E_{\mathrm{mean}}^{\prime }=1773\pm 298\;\text{Pa}$ and the mean loss modulus was $E^{\prime \prime }=354\pm 102\;\text{Pa}$ (Table 3). The per-bead standard deviations over the 40 repetitions are typically a fraction of the mean, indicating stable repeat measurements for both the elastic and viscous components.

**Table 3.** Thickness and viscoelastic parameters for polyacrylamide beads (10 samples, 40 repetitions each).

**#**	**Thk (µm)**	**Std**	**GT (µm)**	** $E_{\mathrm{mean}}^{\prime }(\text{Pa})\pm \text{Std}$**	** $E^{\prime \prime }(\text{Pa})\pm \text{Std}$**
1	27.22	3.58	31.5	1567 ± 200	393 ± 133
2	30.06	0.40	31	1484 ± 51	420 ± 98
3	29.88	0.91	31	1771 ± 77	404 ± 68
4	31.92	0.57	32	2034 ± 34	515 ± 91
5	31.23	0.52	32	1905 ± 26	438 ± 62
6	26.83	1.94	26	1914 ± 222	303 ± 135
7	21.61	1.79	24	1744 ± 232	383 ± 79
8	23.38	0.24	23	1907 ± 20	262 ± 141
9	31.97	0.43	32	2247 ± 47	294 ± 161
10	21.83	0.37	25	1129 ± 22	136 ± 54
**Mean**	**25.12**	–	**27.59**	**1773.20**	**354.80**

It is important to note that the geometric ground-truth (GT) thickness values derived from lateral diameter measurements may not always reflect the true maximum thickness. In Table 3, the 10th bead has a GT thickness of 25μm estimated from its lateral size, whereas our in-line reconstruction yields a maximum thickness of 21.83±0.37μm . Importantly, an independent phase-shifting interferometry (PSI) depth measurement on the same bead reports a maximum thickness of 19.96±0.24μm , supporting the reconstructed value. To further validate this point, we also include direct thickness comparisons between our method and PSI on the same beads in Supplement 1 Figs. S1 and S2, which show close agreement in the recovered 3D surface profiles and cross-sectional depth traces, with residual differences primarily confined to edge regions.

In addition, Table 4 reports the corresponding viscoelastic parameters extracted from MCF-7 cells for reference. While the cell values are expectedly lower than bead measurements, the same reconstruction pipeline produces consistent thickness and KV parameter estimates across the cell cohort, supporting the applicability of the method to softer biological samples.

**Table 4.** Thickness and viscoelastic parameters of MCF-7 cells.

**Cell #**	**t (µm)**	**Std**	** $E_{\mathrm{mean}}^{\prime }(\text{Pa})$ **	**Std**	** $E^{\prime \prime }(\text{Pa})$ **	**Std**
1	11.83	2.01	297.76	104.16	83.95	56.56
2	10.06	1.43	231.21	92.18	109.17	48.98
3	12.19	0.95	341.18	8.60	63.12	14.60
4	7.62	1.18	257.47	39.79	65.06	32.78
5	10.06	0.38	297.98	4.71	43.91	20.07
6	7.04	0.41	154.46	16.96	21.64	12.57
7	8.38	1.80	210.30	78.82	57.34	33.92
8	11.55	0.22	288.05	29.22	37.52	24.21
9	6.41	0.26	133.59	15.98	20.07	9.71
10	8.53	0.30	297.44	7.58	43.80	20.36
11	6.30	0.09	243.89	4.87	29.89	15.81
12	7.24	0.23	249.82	14.20	30.16	23.48
13	5.42	0.26	162.46	7.71	21.98	9.79
14	12.36	0.15	380.92	13.22	51.08	34.22
**Mean**	**8.79**	–	**253.32**	–	**48.47**	–

Finally, the reconstructed bead stiffness values are consistent with our prior off-axis measurements on polyacrylamide beads [29], supporting the quantitative validity of the in-line approach. Overall, these bead experiments confirm that the proposed vibration-encoded in-line framework can stably recover thickness as well as Kelvin–Voigt viscoelastic parameters ( $E^{\prime }$ and $E^{\prime \prime }$ ) under real imaging conditions, providing a calibrated experimental baseline for subsequent cell measurements.

### 3.3. Application to MCF-7 breast cancer cells

The same experimental procedure and reconstruction pipeline were then applied to adherent MCF-7 breast cancer cells to demonstrate applicability to soft biological specimens with unknown mechanical properties. Representative reconstructions are shown in Fig. 4(e–h) (raw hologram, thickness, and Kelvin–Voigt viscoelastic maps $E^{\prime }$ and $E^{\prime \prime }$ ), and the corresponding per-cell summary statistics are reported in Table 4.

Across the measured cohort ( n=14cells, 40 repeats per cell), the reconstructed peak thickness values ranged from t=5.42 to 12.36μm (mean 8.79μm ). The recovered storage modulus spanned $E_{\mathrm{mean}}^{\prime }=133.59–380.92\;\text{Pa}$ (mean 253.32Pa), while the loss modulus ranged from $E^{\prime \prime }=20.07$ to 109.17Pa (mean 48.47Pa ), corresponding to a mean complex modulus magnitude of $|E^{*}|\approx 257.92\;\text{Pa}$ . These values are consistent with the modulus scale of 300-400 Pa reported for MCF-7 cells by Mirzaluo *et al.* [32]; however, whereas that study assumed a purely elastic response, our Kelvin-Voigt-based characterization additionally quantifies the dissipative component, yielding a comparable modulus magnitude while accounting for the intrinsic viscoelasticity of adherent cells.

## 4. Discussion and conclusion

The proposed in-line Mach–Zehnder interferometric approach provides a practical alternative to both off-axis and conventional in-line digital holography for quantitative phase and viscoelastic imaging. In off-axis configurations, the lateral carrier introduced by the reference beam separation inevitably limits the usable spatial bandwidth, leading to a reduction in effective resolution and field of view. Although off-axis holography enables single-shot phase retrieval, this trade-off between spatial frequency coverage and alias-free reconstruction restricts its applicability in high-resolution or wide-field biological imaging.

In contrast, traditional in-line phase-shifting holography preserves the full optical bandwidth but requires multiple recordings at precisely controlled reference phase shifts. This demands sub-wavelength mechanical stability of both the optical system and the specimen throughout the acquisition sequence. Even small temporal drifts or vibrations between successive phase steps can induce substantial reconstruction errors, especially when imaging living or dynamic samples.

Table 5 summarizes the main features of each approach. The system developed in this study overcomes the main limitations of both techniques by using controlled specimen vibration to encode phase modulation directly within a single cycle. Rather than mechanically modulating the reference path, the object’s sinusoidal motion generates a series of intensity holograms with phase steps set by its periodic displacement. Sampling twelve evenly spaced frames per cycle allows direct extraction of both the optical phase and the local vibration-induced modulation depth (*β*). This configuration offers self-referenced phase recovery without external phase shifters or interferometric stabilization.

**Table 5.** Comparison of phase retrieval approaches in digital holography.

**Method**	**Advantages**	**Limitations**
**Off-axis holography**	Single-shot phase retrieval; robust against sample motion; widely used and well established.	Spatial bandwidth reduced due to carrier frequency; limited resolution and field of view; requires angular alignment and spatial filtering.
**In-line phase-shifting holography (PSI)**	Full spatial bandwidth preserved; high phase accuracy when phase steps are precisely controlled.	Requires multiple sequential frames with precise phase delays; highly sensitive to mechanical drift and environmental instability; unsuitable for dynamic samples.
**Proposed vibration-based in-line approach**	Preserves full optical bandwidth; no external phase modulation; enables simultaneous phase and elasticity mapping within a single vibration cycle.	Requires the sample to undergo periodic deformation; applicable primarily to soft materials with monotonic *β* response; temporal resolution limited by vibration frequency and camera frame rate.

Beyond recovering the quantitative phase, the proposed method leverages the measured *β* distribution to infer the local viscoelastic parameters of the sample. Because *β* reflects the phase response under a known driving stress, its spatial variation encodes the mechanical compliance of the object. The only necessary condition is that the sample must be sufficiently deformable such that the vibration amplitude remains within the monotonic region of the *β*–phase response, ensuring a one-to-one relationship between the measured modulation and the underlying stiffness. This condition is readily satisfied for soft polymeric beads, hydrogels, and biological cells, making the method suited for micromechanical characterization of soft matter.

The results presented here confirm that both thickness and elasticity can be retrieved with high quantitative fidelity from simulated and experimental datasets. Compared to reference geometries, the proposed technique combines the wide bandwidth and straightforward alignment of in-line holography with the quantitative rigor of multi-phase interferometry, yet without requiring path modulation or reference-beam stability. Here, reconstruction does not rely on Fourier-domain cropping, spatial-frequency truncation, or off-axis carrier isolation which reduce spatial resolution; instead, the full in-line hologram spectrum supported by the system NA is preserved and used in phase recovery. A standard spatial-resolution validation (e.g., MTF or resolution-target measurement) was not performed because conventional resolution targets are optically rigid and do not exhibit the monotonic *β* response required by the vibration-encoded mechanical readout, while deformable targets with well-defined feature sizes and suitable dynamic response are not commercially available.

Future developments will focus on extending the proposed approach to higher vibration frequencies and faster image acquisition, enabling time-resolved mapping of optical phase and stiffness in living samples. Advances in illumination control, sensor bandwidth, and synchronization are expected to facilitate continuous, real-time mechanical imaging under physiologically relevant conditions. In addition, the use of higher numerical aperture optics may further enhance spatial resolution, allowing subcellular structural features to be visualized alongside their local mechanical properties.

Importantly, the proposed framework is not intrinsically limited to a single actuation modality. Although acoustic excitation is employed in the present study, alternative mechanical stimulation strategies—such as magnetic, optical, or microfluidic actuation—can be incorporated in future implementations. These approaches may offer improved force uniformity and enhanced stability of the induced deformation, thereby relaxing the requirement for strictly periodic motion and monotonic *β* behavior. In addition, non-contact actuation schemes eliminate the need for direct mechanical coupling to the substrate. This flexibility is expected to broaden the applicability of the method.

## Supplemental information

Dataset 1 Time-series interferometric images capturing the vibrational response of a polyacrylamide bead https://doi.org/10.6084/m9.figshare.30990529

Supplement 1 Supplement 1 https://doi.org/10.6084/m9.figshare.31077502

## Data Availability

The data and the code that support the findings of this study are available from the corresponding author upon request. The raw bead hologram sequence used for Fig. 4(a) is provided as Dataset 1 [31].

## Supplemental document

See Supplement 1 for supporting content.
