# Supplementary material for: Inline mechano-vibration holography for simultaneous phase and elasticity mapping of soft samples
Source: Biomed Opt Express. 2026 Jan 23;17(2):901–15. doi: 10.1364/BOE.584264 (PMC12904524; doi:10.1364/BOE.584264)
Supplement: Supplementary file 1 [file boe-17-2-901-s001.pdf]

Supplemental Document

Biomedical Optics EXPRESS

Inline mechano-vibration holography for simultaneous phase and elasticity mapping of soft samples: supplement

HASAN BERKAY ABDIOGLU,[1] YAGMUR ISIK,[1] MERVE SEVGI,[2] ESMAHAN CAGLAR,[2] GOKHAN BORA ESMER,[3] HUSEYIN UVET,[1,4,6] AND ALI ANIL DEMIRCALI[5,7,]

[1]*Yildiz Technical University, Department of Mechatronics Engineering, Istanbul 34349, Turkey*
[2]*Yildiz Technical University, Department of Bioengineering, Istanbul 34349, Turkey*
[3]*Yildiz Technical University, Department of Control and Automation Engineering, Istanbul 34349, Turkey*
[4]*Istinye University, Artificial Intelligence Research and Application Center (YZAUM), Istanbul 34467, Turkey*
[5]*Imperial College London, Division of Systems Medicine, Department of Metabolism, Digestion, and Reproduction, London SW7 2AZ, UK*
[6]*huvet@yildiz.edu.tr*
[7]*a.demircali@imperial.ac.uk*

This supplement published with Optica Publishing Group on 23 January 2026 by The Authors under the terms of the Creative Commons Attribution 4.0 License in the format provided by the authors and unedited. Further distribution of this work must maintain attribution to the author(s) and the published article's title, journal citation, and DOI.

Supplement DOI: https://doi.org/10.6084/m9.figshare.31077502

Parent Article DOI: https://doi.org/10.1364/BOE.584264

Inline Mechano-Vibration Holography For Simultaneous Phase And Elasticity Mapping of Soft Samples

Hasan Berkay Abdioglu, Yagmur Isik, Merve Sevgi, Esmahan Caglar, Gokhan Bora Esmer, Huseyin Uvet* and Ali Anil Demircali*

Contents:

Supplementary Figure S1: A microspherical semitransparent object (Polyacyrlamide bead) thickness and phase map measured by phase shifting holography (PSI) and Acoustic Phase Shifting Method (APS)

Supplementary Figure S2: A microspherical semitransparent object (Polyacyrlamide bead) thickness and phase map measured by phase shifting holography (PSI) and Acoustic Phase Shifting Method (APS)

Supplementary Table S1: Reconstruction error of APS under angular (alignment) jitter applied over one vibration cycle. Reported metrics are RMSE (and standard deviation across trials) for phase-lag error, viscosity error, and elastic-modulus error within a fixed mask.

Supplementary Table S2: Reconstruction error of APS under phase jitter (sampling-phase uncertainty) applied over one vibration cycle. Reported metrics are RMSE (and standard deviation across trials) for phase-lag error, viscosity error, and elastic-modulus error within a fixed mask.

Supplementary Table S3: Ablation study of regularization components in the APS pipeline. Each row removes one processing step and reports the resulting mean Kelvin–Voigt parameters and reconstruction errors (RMSE of elastic modulus, viscosity, and phase lag) relative to the full pipeline.

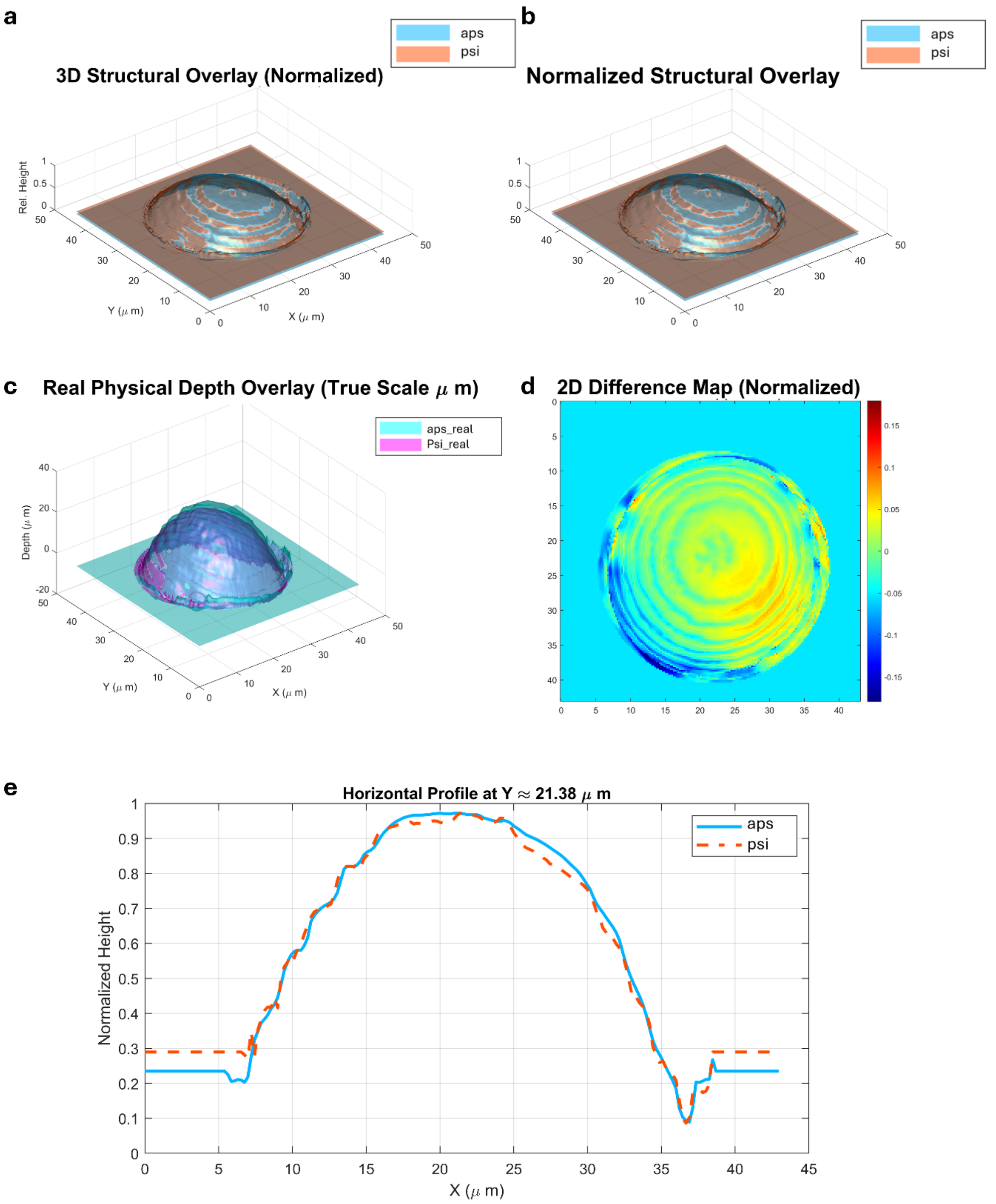

Supplementary Fig. S1. Detailed comparison between acoustic phase shifting (APS) and conventional phase-shifting interferometry (PSI) for phase-based surface reconstruction. (a) Normalized three-dimensional surface reconstructed using APS and (b) normalized three-dimensional surface reconstructed using PSI, showing comparable global morphology. (c) Three-dimensional surface profiles in physical depth units (μm), demonstrating strong overlap in the recovered height distribution; the dominant discrepancy appears as a near-constant depth offset rather than a change in shape. (d) Normalized two-dimensional difference map (APS minus PSI), indicating that residual differences are primarily localized near edge regions and

high-gradient areas. (e) Representative horizontal cross-sectional profiles, confirming close agreement in surface shape between APS and PSI. The observed global offset is attributed to differences in phase referencing and zero-baseline definition (e.g., unwrapping/edge grounding), rather than structural disagreement.

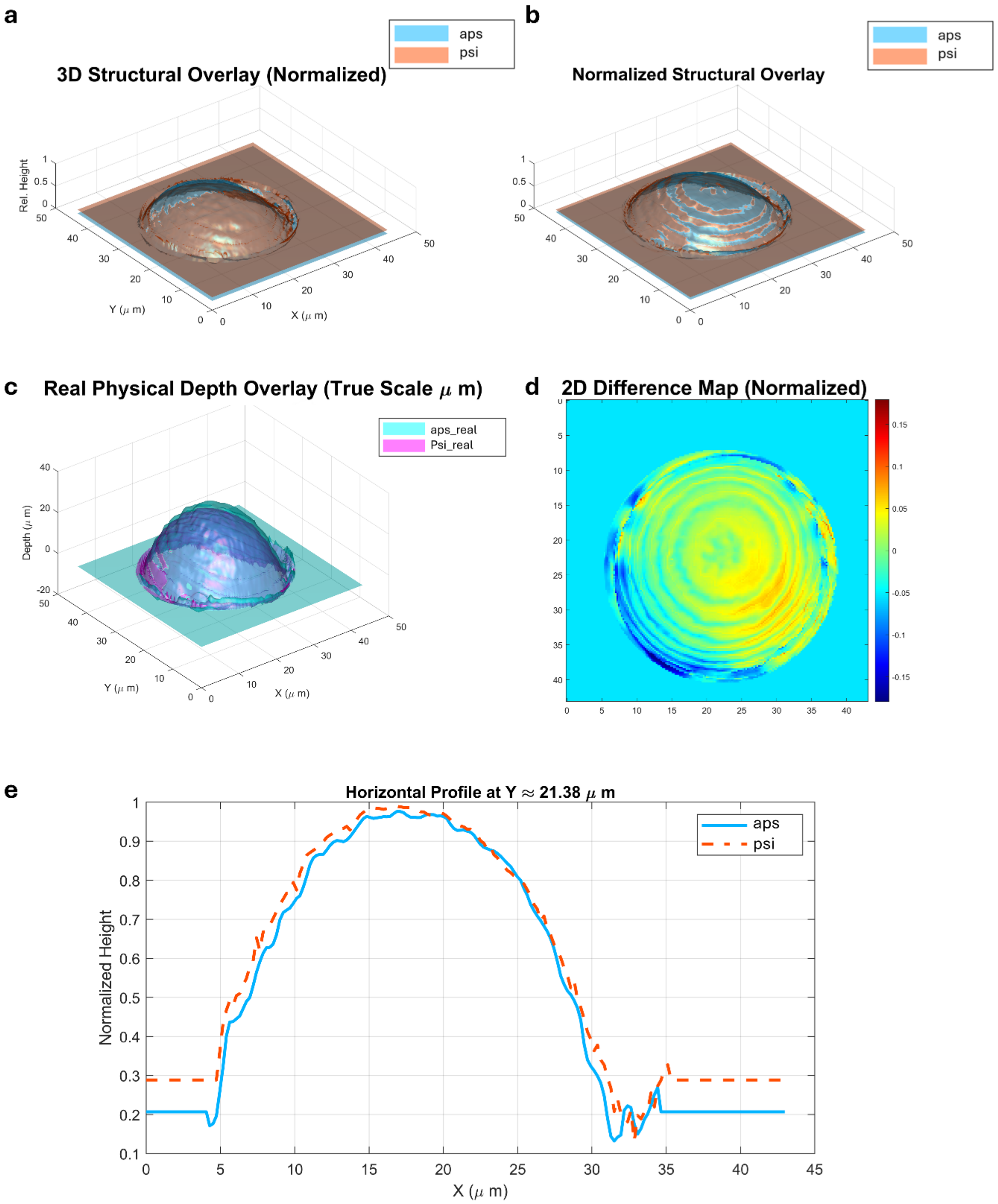

Supplementary Fig. S2. Reconstruction agreement is consistent with Fig. S1, confirming reproducibility across bead samples.

Supplementary Table S1: APS reconstruction RMSE under angular (alignment) jitter over one vibration cycle.

Angle jitter σθ (deg)	δ error RMSE on fixed mask (deg)	SD(δ RMSE)	η error RMSE on fixed mask (Pa·s)	SD (η RMSE)	E error RMSE on fixed mask (Pa)	SD (E RMSE)
0.00	0.028516374	3.88E-18	0.017098543	0	17.502425	0.00000000
0.01	0.042634624	1.64E-02	0.017672373	0.000392295	17.936495	0.58534091
0.02	0.113269098	4.10E-02	0.018259895	0.001881805	17.924025	0.87819882
0.03	0.175583613	7.42E-02	0.078778414	0.132913905	86.596396	153.82624600
0.05	0.278879046	8.88E-02	0.039648821	0.016536621	37.699517	20.59739208
0.07	0.217103805	4.02E-02	0.069191834	0.064703074	74.127936	75.68181149
0.10	0.505050098	2.40E-01	0.074756378	0.052153109	85.267177	64.61037327
0.12	0.504468519	1.90E-01	0.033734571	0.001168755	25.733280	5.47523542
0.15	0.770517465	3.76E-01	0.111172500	0.069568665	109.702914	110.02295060
0.20	0.888565712	6.53E-01	0.086387443	0.077458205	84.799732	99.87174709
0.30	0.962003744	2.92E-01	0.129615779	0.099921553	130.853941	126.45000900
0.40	1.596750726	8.64E-01	0.180690348	0.047251315	165.573669	85.02529453
0.50	2.650997176	9.75E-01	0.282960270	0.125513323	288.247843	165.31386890
0.70	3.730326137	2.22E+00	0.215524743	0.098427696	157.956462	56.73825278
1.00	3.657540285	1.67E+00	0.253664018	0.107669719	259.941094	61.40714789
1.30	5.829960479	3.22E+00	0.473972104	0.056237769	441.411391	86.33052631
1.60	6.856890765	3.23E+00	0.570950606	0.204041996	538.882474	346.81718130
2.00	7.064454384	4.72E+00	0.546812858	0.140748846	668.809463	193.90693020

Supplementary Table S2: APS reconstruction RMSE under phase jitter over one vibration cycle.

Phase jitter σφ (rad)	δ error RMSE on fixed mask (deg)	SD(δ RMSE)	η error RMSE on fixed mask (Pa·s)	SD(η RMSE)	E error RMSE on fixed mask (Pa)	SD(E RMSE)
0.000	0.028516374	3.88E-18	0.017098543	0	17.5024245	0.00000000
0.001	0.398127473	1.87E-01	0.203444381	0.051269464	215.8130562	63.74177598
0.002	1.373164344	4.33E-01	0.187833444	0.065264902	181.3015972	92.34992730
0.003	0.870645377	4.75E-01	0.193392530	0.125374296	183.9847236	116.38130130
0.005	2.675735943	1.70E+00	0.257342033	0.106937584	247.1607392	123.52394850
0.007	2.926882526	2.09E+00	0.224957433	0.092715742	175.0036114	100.42094150
0.009	3.078390756	2.82E+00	0.276007087	0.070822351	210.5453642	81.55023174
0.010	4.617337341	2.13E+00	0.310932281	0.062403866	328.4134767	140.09902340
0.012	4.698848247	2.63E+00	0.347578017	0.115345325	346.6942119	81.21770492
0.015	3.545957775	2.87E+00	0.280275796	0.079093668	340.1551325	89.40627376
0.020	6.409889359	1.91E+00	0.311216707	0.137059027	318.9991624	133.93171560
0.025	6.854690004	3.55E+00	0.518691327	0.124058601	476.4844142	117.95950320
0.030	11.864783170	8.45E+00	0.595298873	0.326820847	647.1690963	323.03892570
0.040	12.285093610	8.46E+00	0.569545780	0.162353092	616.0087245	106.36585900
0.050	15.108719010	1.08E+01	0.720322446	0.051153741	718.9812885	116.07267200
0.070	14.224300090	6.35E+00	0.689599006	0.134174435	774.7655250	152.17987540
0.100	16.541587740	1.16E+01	0.784531555	0.161377541	769.8627163	90.89847383
0.150	19.001050280	1.00E+01	0.789032111	0.133003393	961.4285796	243.90249090
0.200	25.773036110	1.51E+01	0.789265824	0.079962824	965.0920190	139.52077470
0.250	22.807872300	1.00E+01	0.837638655	0.192441074	996.3870624	195.69108540
0.300	19.655564270	9.10E+00	0.915275872	0.102753872	1172.2557320	228.49489590

Supplementary Table S3: APS pipeline ablation study: effect of removing regularization steps on reconstructed Kelvin–Voigt parameters and RMSE.

Case	E_mean	eta mean	delta_mean deg	E_RMSE	eta_RMSE	delta_RMSE deg
FULL (all regs)	12101	1.2022	31.972	20.827	0.020687	0.000271
No outer annulus ref	1210.1	1.2022	31.972	20.827	0.020687	0.000271
No rim baseline	1120.0	1.1127	31.972	85.308	0.084752	0.000271
No phiT clamp	1210.1	1.2022	31.972	20.827	0.020687	0.000271
No delta smooth	1251.1	1.2429	31.972	58.323	0.057937	0.000213
No beta-floor	1210.1	1.2022	31.972	20.827	0.020687	0.000271
No ring-exclude	1184.4	1.1184	29.728	92.146	0.123910	3.450500
No refined mask	1210.1	1.2022	31.972	20.827	0.020687	0.000271
No sign-fix	1210.1	1.2022	31.972	20.827	0.020687	0.000271
Mean instead of median	1227.6	1.2195	31.972	33.938	0.033711	0.000271
