# Supplementary figures and images for: Inline mechano-vibration holography for simultaneous phase and elasticity mapping of soft samples
Source: Biomed Opt Express. 2026 Jan 23;17(2):901–15. doi: 10.1364/BOE.584264 (PMC12904524; doi:10.1364/BOE.584264)

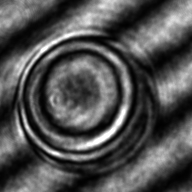

Supplement: Supplementary file 2 [file boe-17-2-901-v001.tar › PAA_single_cycle/frame_0001.tif]

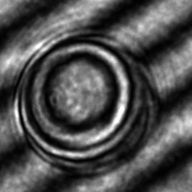

Supplement: Supplementary file 2 [file boe-17-2-901-v001.tar › PAA_single_cycle/frame_0002.tif]

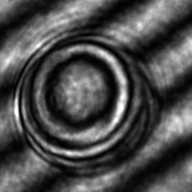

Supplement: Supplementary file 2 [file boe-17-2-901-v001.tar › PAA_single_cycle/frame_0003.tif]

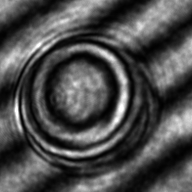

Supplement: Supplementary file 2 [file boe-17-2-901-v001.tar › PAA_single_cycle/frame_0004.tif]

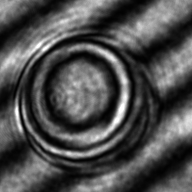

Supplement: Supplementary file 2 [file boe-17-2-901-v001.tar › PAA_single_cycle/frame_0005.tif]

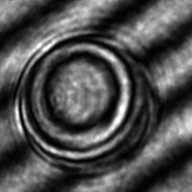

Supplement: Supplementary file 2 [file boe-17-2-901-v001.tar › PAA_single_cycle/frame_0006.tif]

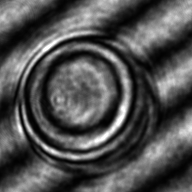

Supplement: Supplementary file 2 [file boe-17-2-901-v001.tar › PAA_single_cycle/frame_0007.tif]

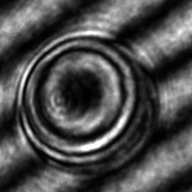

Supplement: Supplementary file 2 [file boe-17-2-901-v001.tar › PAA_single_cycle/frame_0008.tif]

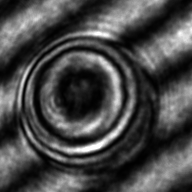

Supplement: Supplementary file 2 [file boe-17-2-901-v001.tar › PAA_single_cycle/frame_0009.tif]

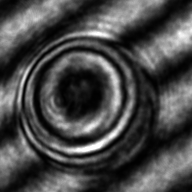

Supplement: Supplementary file 2 [file boe-17-2-901-v001.tar › PAA_single_cycle/frame_0010.tif]

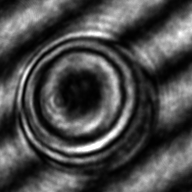

Supplement: Supplementary file 2 [file boe-17-2-901-v001.tar › PAA_single_cycle/frame_0011.tif]

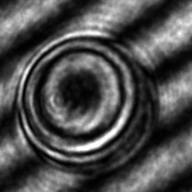

Supplement: Supplementary file 2 [file boe-17-2-901-v001.tar › PAA_single_cycle/frame_0012.tif]
